# A Medical Causerie

**Published:** 1916-06-03

**Authors:** 


### A MEDICAL CAUSERIE.

But for the clause introduced into the Military Service Bill by the House of Lords, on the proposal of the Government, medical practitioners of military age—that is, of less than forty-one years—would now be in relation to the Army in exactly the same position as their non-medical fellow-citizens. They would be called upon to take combatant service with the Colours, unless declared physically unfit or exempted by the local tribunal. But the Army does not want doctors in the combatant ranks; it wants them in their professional capacity. Hence the new clause provides for the institution of professional committees and the reference to these committees of all appeals for exemption from service by medical practitioners. In other words, every medical practitioner of less than forty-one years of age, so soon as the new Act comes into operation, is compelled to join the Army as a medical officer unless he can convince the duly constituted professional committee that he ought to be granted a certificate of exemption. There is not yet any official announcement on the subject, but it is morally certain that the professional committees to be recognised for this purpose will be the English, Scottish, and Irish Medical War Committees. Hence, whether the doctor of military age enrols or does not enrol with one or other of the medical committees, it is with such a committee that the decision of his duty will lie, for even should he put his case in the first place to the local tribunal, this tribunal is bound to refer the matter to the professional authority, and to accept the finding which this authority adopts. Those practitioners who, though of military age, refused voluntary adhesion to the scheme of enrolment, now therefore find themselves, under the pressure of the law, in exactly the position they declined to accept on the invitation of the Central Medical War Committee. Whether they must join the Army or must remain in civil practice will be settled not by their individual choice, but by the Medical War Committee. The task thus falling on the War Committee is a very delicate one, but it cannot be avoided. Doctors are essential both to the Army and to the civil population, and the supply of doctors is a limited one. The question, therefore, is one of organisation and distribution, and the general principles which must guide decision have already been fairly well determined. The work done during the last twelve months or so will now prove extremely valuable, and amongst those who will benefit by it will be doctors who have to leave their practices for military duties. Arrangements to protect the interests of the absent practitioners have been well organised, and will no doubt command loyal support.

---

Truly a touching belief in the power of medicine was displayed by the convict who recently begged the Court of Criminal Appeal to order him to be put under chloroform and to be questioned when fully anæsthetised. Apparently his notion was that what he said in these circumstances would have the guarantee of truth, and that being innocent he would announce this fact and would be believed. Perhaps the truth does sometimes come out under chloroform, but not always, it is to be hoped.

---

Many of the stories told of the attempts of medical practitioners to deal in a non-committal fashion with the difficulties of prognosis have a frivolous and sometimes a gruesome quality, and are apt to jar on serious minds. This in some degree is the position of the reply attributed to a distinguished physician who was asked by a noble lady whether her sick servant would live through the night, and who, it is said, answered solemnly, "I will tell your ladyship in the morning." Recently in one of the American journals a house physician, in recounting his experiences, boasted that in one of his prognostic efforts he had been very nearly right, for though the patient for whom he had prophesied an early and fatal issue made as a fact a good recovery, the patient in the next bed died the same night! One of the most successful efforts to be provided for any event is surely that of the doctor who when consulted in a case of suspected insanity replied, "I do not say the man is mad in the ordinary sense of this term, but neither do I say that it is prudent he should be left at liberty."

---

The Daylight Saving Act, as indeed is the case with many legislative experiments, will probably produce some unexpected consequences. Already it is alleged to be responsible in several directions for evil results. Thus the farmers who would fain have their labourers at work by the clock find this to be impossible, for the grass and hay remain wet with dew to the usual time; children refuse to be cheated into going to bed, seeing that the daylight says it is not bedtime, and hence they lose sleep and invade the quiet evening of their parents, not to add that their moral sense is weakened by the admittedly erroneous announcement of the parish clock; electric-light companies say that to compensate for diminished consumption they must raise prices; and, again, non-patriotic motorists take evening runs and thus waste petrol required by the country. But there is yet a possibility of far more serious import, and it has been discovered by a Peer of the realm. Suppose, it is said, that on the night of September 30 a woman gave birth to twins, that one of these was born at 12.50 and the other ten minutes later. But at one o'clock by law the hands of the clock would be put back to midnight. Hence, while the first-born would be registered officially as arriving in the world at 12.50 the child who appeared later would be marked as born at 12 o'clock. The possibilities might be serious in reference to the inheritance of, say, money or a title. Yet it looks as if we shall have to take the risk such as it is.

